# Snake-Eye Myelopathy and Surgical Prognosis: Case Series and Systematic Literature Review

**DOI:** 10.3390/jcm9072197

**Published:** 2020-07-12

**Authors:** Marco Maria Fontanella, Luca Zanin, Riccardo Bergomi, Marco Fazio, Costanza Maria Zattra, Edoardo Agosti, Giorgio Saraceno, Silvia Schembari, Lucio De Maria, Luisa Quartini, Ugo Leggio, Massimiliano Filosto, Roberto Gasparotti, Davide Locatelli

**Affiliations:** 1 Neurosurgery Unit, Department of Medical and Surgical Specialties, Radiological Sciences and Public Health, University of Brescia, 25123 Brescia, Italy; lucazanin00@gmail.com (L.Z.); rbergomi@tiscalinet.it (R.B.); costanzamaria.zattra01@universitadipavia.it (C.M.Z.); g.saraceno@unibs.it (G.S.); luciodemaria@gmail.com (L.D.M.); 2 Neurosurgery Unit, Poliambulanza Foundation, 24124 Brescia, Italy; marcofaziomd@gmail.com; 3 Neurosurgery Unit, Department of Biotechnology and Life Sciences (DBSV), University of Insubria, Ospedale di Circolo e Fondazione Macchi, 21100 Varese, Italy; edoardo_agosti@libero.it (E.A.); silvia.schembari@outlook.com (S.S.); davide1.locatelli@uninsubria.it (D.L.); 4 Intensive Care Unit, Department of Anesthesia, Intensive Care and Emergency, ASST Spedali Civili di Brecia, 25123 Brescia, Italy; neuro.chirurgiabs@gmail.com; 5 Neurophysiopathology Unit, Department of Neurological Sciences and Vision, ASST Spedali Civili di Brecia, 25123 Brescia, Italy; ugo.leggio@unibs.it; 6 Center for Neuromuscular Diseases, Unit of Neurology, ASST “Spedali Civili”, 25123 Brescia, Italy; massimiliano.filosto@unibs.it; 7 Neuroradiology Unit, Department of Medical and Surgical Specialties, Radiological Sciences, and Public Health, University of Brescia, 25123 Brescia, Italy; roberto.gasparotti@unibs.it

**Keywords:** snake-eye, owl sign, Hirayama disease, degenerative cervical myelopathy (DCM)

## Abstract

The prognostic value of “snake-eyes” sign in spinal cord magnetic resonance imaging (MRI) is unclear and the correlation with different pathological conditions has not been completely elucidated. In addition, its influence on surgical outcome has not been investigated in depth. A literature review according to PRISMA (Preferred reporting items for systematic review and meta-analysis protocols) guidelines on the prognostic significance of “snake-eyes” sign in operated patients was performed. Clinical, neuroradiological, and surgical data of three institutional patients, were also retrospectively collected. The three patients, with radiological evidence of “snake-eyes” myelopathy, underwent appropriate surgical treatment for their condition, with no new post-operative neurological deficits and good outcome at follow-up. The literature review, however, reported conflicting results: the presence of “snake-eyes” sign seems a poor prognostic factor in degenerative cervical myelopathy, even if some cases can improve after surgery. “Snake-eyes” myelopathy represents a rare form of myelopathy; pathophysiology is still unclear. The frequency of this myelopathy may be greater than previously thought and according to our literature review it is mostly a negative prognostic factor. However, from our experience, prognosis might not be so dire, especially when tailored surgical intervention is performed; therefore, surgery should always be considered and based on the complete clinical, neurophysiological, and radiological data.

## 1. Introduction

The “snake-eyes” appearance (SEA) or sign, also referred to as “owl-eyes” or “fried-eggs” sign, is a unique radiological finding appearing as bilateral hyperintense symmetric, circular or ovoid foci on T2-weighted (T2W) axial magnetic resonance imaging (MRI) sequences in the anterior horn cells of the spinal cord. It was first reported by Jenkins and Al-Mefty in 1986 [1]. The prognostic significance of this radiological finding has been debated in several articles with conflicting results [2,3,4,5].

SEA appearance is described in association with several clinical conditions like anterior spinal artery ischemia [6], chronic compressive myelopathy [7], degenerative cervical myelopathy (DCM) [4], Hirayama disease [8,9] or monomelic amyotrophy of the upper limb, amyotrophic lateral sclerosis [10], and spinal muscular atrophy [11]. The relationship between these ailments and the pathophysiology of SEA is not totally clear at present. It has been speculated that SEA is a reversible condition [3]. This claim is in contrast with its histopathology: in fact, SEA is the result of cystic necrosis at the junction of the central grey matter near the ventrolateral posterior column [12].

There is, therefore, a need to better understand SEA prognostic significance, and especially its influence on surgical outcome [13]. In this study, we evaluated the prognostic role of SEA through a systematic literature review and an analysis of our most recent patients who underwent surgical treatment. 

## 2. Experimental Section

### 2.1. Literature Review

The systematic review of the literature was performed in March 2020 according to PRISMA guidelines [14]. Synthesis Without Meta-analysis (SWiM) guidelines were applied [15].

PubMed, Ovid MEDLINE, and Ovid EMBASE databases were searched using the keywords: “snake-eye myelopathy”, “owl-eye myelopathy”, “fried-eggs sign”, “snake-eye appearance”, “owl-eye appearance”, and their variations. English studies published between December 1989 and December 2019 were included. 

Inclusion criteria were: (1) studies with description of MRI-evident SEA myelopathy and surgery; (2) studies concerning a specific pathology related to SEA myelopathy, with patients undergoing surgical treatment; (3) studies with a clinical follow-up of surgically treated SEA patients. Exclusion criteria were: (1) absence of prognostic results about “snake-eyes sign”; (2) other radiological findings than SEA myelopathy; (3) absence of long-term follow-up. 

For each study, we extracted the following baseline information: type of study, number of cases, clinical background, and prognostic value. The primary endpoint of the review was clinical outcome following surgical treatment in patients with SEA myelopathy.

### 2.2. Case Series

We reviewed data of all our patients with a diagnosis of DCM, who underwent surgical treatment over the past year. Patients were included in our study if (1) they had a record of “snake-eye” sign on their T2W MRI sequences and they (2) gave consent to use of their information for research purposes. 

Recorded information included: baseline demographic and clinical data (age at presentation and gender; symptoms and signs at presentation), treatment strategy, outcome at discharge and follow-up. Modified Japanese Orthopedic Association Score (mJOA score) [16] and Medical Research Council (MRC) Muscle Scale [17] were adopted for pre- and post-operative neurological evaluation.

## 3. Results

### 3.1. Literature Review

**Scheme 1:**
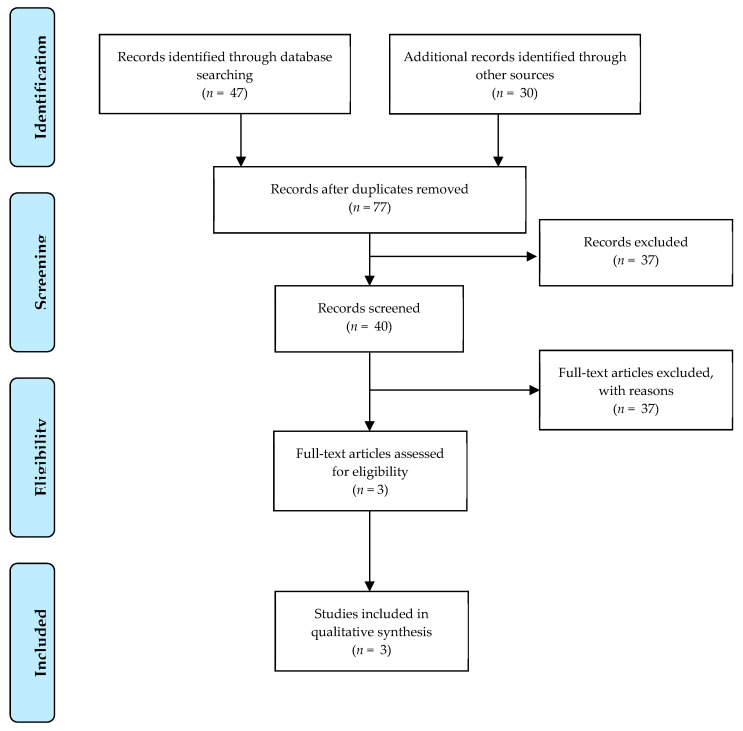
Literature review process, according to PRISMA guidelines.

A total of 77 papers were identified after duplicates removal. After title and abstract analysis, 40 articles were identified for full-text analysis. Eligibility was ascertained for three articles. PRISMA flow chart is shown in Scheme 1. SWiM scheme is reported in Table 1.

Choi (2005) [4] reported 47 retrospective cases of DCM, Mizuno (2003) [12] described a total of 144 retrospective cases of degenerative cervical myelopathy with a mean pre-operative mJOA score of 10.8, and Zhang (2010) [2] reported 106 retrospective cases with a diagnosis of DCM with a mean pre-operative mJOA of 8.70. 

In detail, subgroup analysis reported a total of 81 patients with ossification of posterior longitudinal ligament (OPLL).

Regarding DCM, the “snake-eye” appearance was regarded as a negative prognostic factor in 144 cases (48.5%). In particular, in Mizuno’s study, the improvement ratio determined by the JOA score was 32.2% in SEA (mean post-operative mJOA score of 12.9), 47.1% in NSEA, and 50% (*p* < 0.01) in control cases in which high signal intensity was absent.

### 3.2. Case Series 

**Figure 1:**
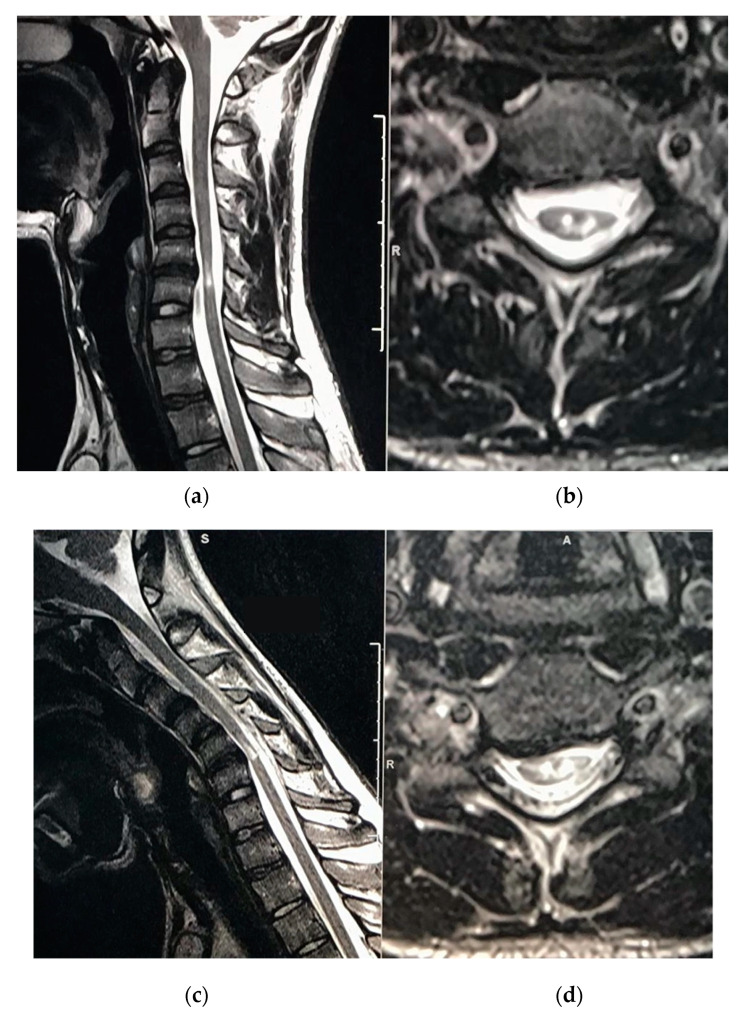
Dynamic sagittal and axial cervical T2-MRI scan: (**a**) sagittal T2-MRI scan with visible myelopathy at C5–C6 level; (**b**) axial T2-MRI scan with an already visible SEA appearance; (**c**) sagittal T2-MRI scan in flexion, showing reduced spinal cord canal diameter, with subsequent spinal cord compression; (**d**) axial T2-MRI scan in flexion showing SEA at C5–C6 level and spinal cord compression.

Case 1. A 21-year-old man presented with a one-year history of numbness in the upper limbs with severe loss of hand sensation, especially on the right side. The neurological examination documented upper extremities hypoesthesia and pain, especially in the right arm, with no clear root distribution (mJOA upper extremity sensory subscore 1 of 3) and mild weakness of wrist extensor muscles (MRC grade 4/5), again worse on the right side, with sporadic dropping of objects (mJOA upper extremity motor subscore 4 of 5). No other neurological deficits were detected with a total mJOA score of 15/18. His past medical history included a traumatic injury secondary to hyperflexion of the cervical spine, causing transient acute tetraplegia and distal sensory loss, when he was 4 years old. Cervical spine MRI documented SEA at C5–C6 without stenosis of the vertebral canal. Dynamic flexion MRI showed reduction in the spinal canal diameter with subsequent medullary compression, especially at the C5–C6 level (Figure 1). 

**Figure 2:**
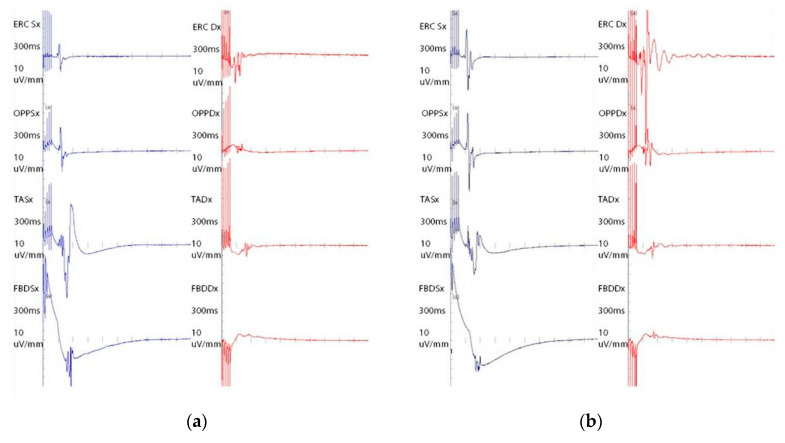
Intra-operative motor evoked potentials (MEPs) prior (**a**) and following (**b**) stabilization. electromyography (EMG) needles record from a hemisphere of about 1 mm. Within this volume there are some 100 muscle fibers. EMG amplitude is the sum of the electric potential differences within a muscle relating to all the active motor units in the vicinity of the electrodes on the skin [18]. Trans-cranial stimulation was performed to elicit MEPs recorded with sub-dermal needle electrodes placed bilaterally in the biceps brachii (BB), extensor radialis carpi (ERC), opponens pollicis (OPP), tibialis anterior (TA), and flexor digitorum brevis (FBD). Prior to stabilization, MEPs were elicited from all monitored muscles on the right side (red) and only from BB, ERC, and TA on the left side (blue) because of technical reasons. Following stabilization, MEPs were essentially unchanged.

An electromyography (EMG) of the upper limbs showed signs of bilateral chronic motor axonal neuropathy in C6 myotome; low amplitude pre-operative motor evoked potentials (MEPs) were detected. The patient underwent an anterior discectomy and fusion at the C5–C6 level with an intersomatic cage. Intra-operative MEP monitoring was performed (Figure 2).

The post-operative clinical course was uneventful with no evidence of new neurological deficits. At the post-operative neurological examination, the patient had a mJOA score of 17 over 18, with an upper extremity motor improvement of 1 point and an upper extremity sensory improvement of 1 point. Wrist extensor muscles weakness resolved completely (MRC grade 5/5). 

**Figure 3:**
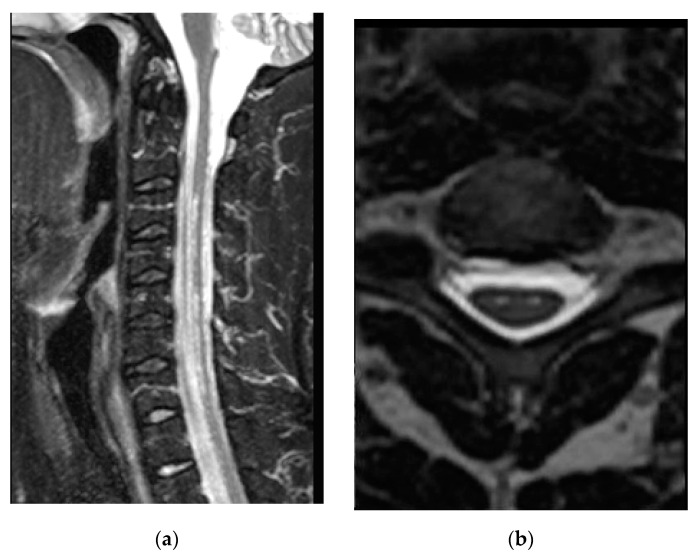
Pre-operative cervical T2-MRI scan: (**a**) sagittal view, showing an anterior pseudomeningocele extending from C3 to T5 with subsequent central canal stenosis and T2-high cord signal consistent with myelomalacia; (**b**) axial view, showing the edematous and T2-hyperintense anterior grey columns, with the characteristic snake-eyes sign.

Case 2. A 44-year-old man suffered a severe traumatic brain injury that required decompressive craniectomy and subsequent cranioplasty. Years later he developed arm cramps, and he was subjected to a cervical MRI scan showing a post-traumatic anterior pseudomeningocele extending from C2 to C5. He underwent multiple lumbar punctures for cerebrospinal fluid (CSF) drainage and even a spinal-peritoneal shunt, which temporarily improved his symptoms, as previously suggested [14]. However, after some time, the pain recurred, along with progressive diparesis (MRC grade 3/5 for both proximal and distal movements) and hypoesthesia, which severely affected his quality of life. Neurological evaluation detected a mJOA score of 13/18 (2/5 upper motor extremity subscore, 1/3 upper sensory extremity subscore). A new MRI scan showed an extension of the already known pseudomeningocele and a new-onset cervical snake-eyes myelopathy at the C5–C6 level (Figure 3). 

**Figure 4:**
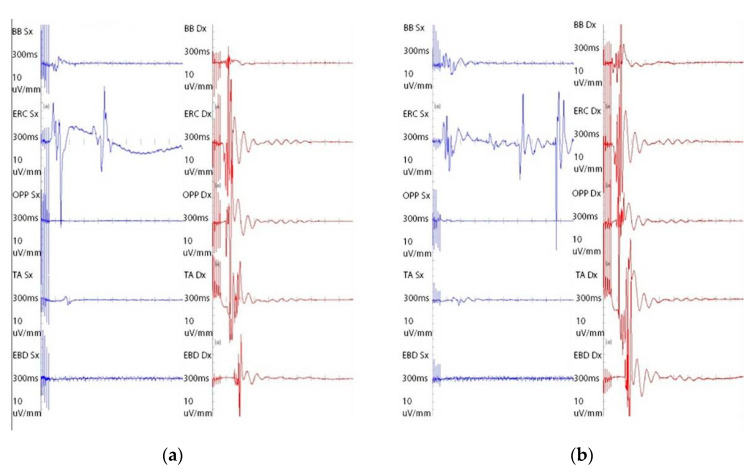
Intra-operative motor evoked potentials (MEPs) prior (**a**) and following (**b**) stabilization. Transcranial stimulation was performed to elicit MEPs recorded with sub-dermal needle electrodes placed bilaterally in the extensor radialis carpi (ERC), opponens pollicis (OPP), tibialis anterior (TA), and flexor digitorum brevis (FBD). Prior to stabilization lower amplitude MEPs were elicited on the right side (red) compared to the left side (blue). Following stabilization higher amplitude MEPs were elicited from the upper limbs especially on the right side.

Superficial upper extremities EMG confirmed denervation in the upper of both arms and low arm myotomes; low-amplitude pre-operative MEPs were detected. It was decided to perform a C3–C7 spine posterior decompression and stabilization. During surgery the patient’s intraoperative MEP did not show any worsening compared to the preoperative ones (Figure 4).

Surgery was uneventful, and, at six months outpatient follow-up, the patient regained significant strength in his arms, especially distally with a MRC grade 4/5 and a mJOA score of 16/18, gaining two points on the upper limbs motor scale. 

**Figure 5:**
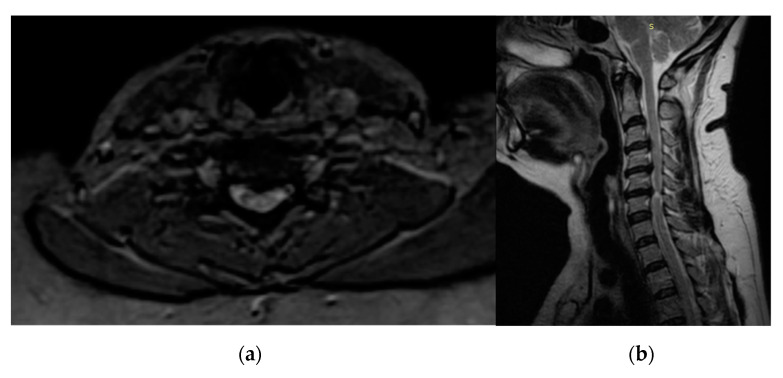
Pre-operative cervical T2-MRI scan: (**a**) axial view, showing cervical spondylosis with associated snake-eyes myelopathy at C5–C6 level; (**b**) sagittal view, showing the cervical canal stenosis with compression of the spinal cord.

Case 3. A 56-year-old woman reported pain in both arms radiating to her hands, especially on the right side, for about five months. The patient’s medical history was unremarkable. She denied recent or past trauma. More recently, she also reported the development of grip loss in her right hand, which affected her daily activities. Neurological examination detected weakness of distal right arm movements (MRC grade 4/5) with mJOA score of 15/18 (upper extremity motor subscore 4/5, upper extremity sensory subscore 1/3). Cervical MRI scan showed cervical spondylosis with associated snake-eyes myelopathy at C5–C6 level (Figure 5). 

**Figure 6:**
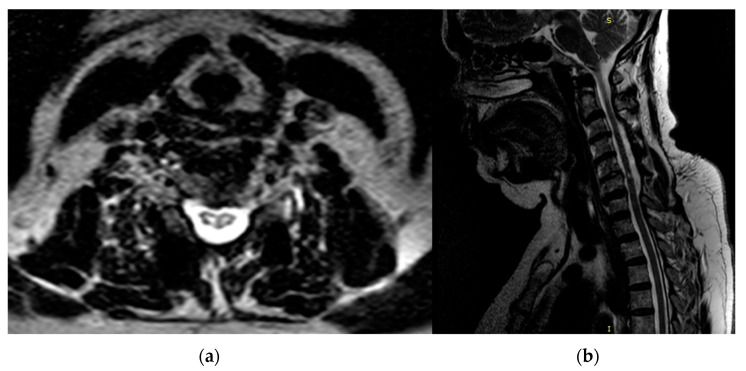
Post-operative cervical T2-MRI scan: (**a**) axial view through the C5–C6 level, showing resolution of the spinal canal stenosis, with persistence of the SEA signal; (**b**) sagittal view, showing resolution of the spinal cord compression.

The patient underwent a C3–C6 laminectomy and cervical arthrodesis. Surgery was uneventful. At discharge pain was reduced, especially in the right arm. The strength in her right hand was completely recovered (MRC grade 5/5 for distal arm movements) at the six month outpatient follow-up, with a mJOA score of 17/18. Post-op MRI scan is shown in Figure 6.

## 4. Discussion

SEA was first presented in a computer tomography (CT) myelography study of seven DCM patients in 1986 [1]. Subsequent anatomopathological studies confirmed that the main modifications were cystic necrosis at the junction of the central grey matter and the posterior ventrolateral column, combined with cell loss in the anterior horn [12]. SEA was reported in other forms of myelopathy too. A clinical randomized trial [8] showed that SEA myelopathy appears during the late stage of Hirayama disease, considered as an anterior horn disorder resulting from local ischemia, triggered by arterial compression from an anterior shifting of the posterior cervical dura upon neck flexion [12].

Undoubtedly, chronic mechanical compression and vascular insufficiency can be among the main promoters of SEA [8,18]. However, the pathogenetic mechanism is not completely understood and research is still ongoing. Although there is no clear data about the exact prevalence and incidence of SEA, some studies suggest that it is much more common than it might be believed [5,8,12].

A thorough literature review shows an inconsistency of results about the prognostic significance of SEA in surgical and non-surgical patients.

According to some studies, SEA does not affect the prognosis of patients who underwent corpectomy and fusion for treatment of DCM [4,5]. Another literature review stated that intense T2W SEA is associated with poorer surgical outcome in patients with DCM, while T2W SEA post-operative regression correlates with better functional outcomes [13]. No report about T1W hypointensity is reported about SEA

Li and Remmel stated that SEA is an irreversible lesion and a predictor of poor prognosis [19]. Mizuno et al. [12] assumed that SEA is an unfavorable prognostic factor for the recovery of upper extremity motor strength and that this is related to neuronal loss in the anterior horn.

A 2015 literature review [20] suggested that SEA can help in the differential diagnosis of spinal cord ischemia, indicating anterior horns infarction caused by anterior spinal artery ischemia.

The pathogenesis of the “snake-eye” myelopathy might be a matter of some debate, but it is interesting that a cervical hypermobility could lead to an anterior compression in flexion, in the absence of a spinal canal stenosis and compression in a neutral position. The institutional Case 1 seems to show this pathogenic mechanism: a dynamic MRI study was useful for a correct therapeutic decision. The surgical strategy, in this type of patient, might be a valid choice.

Regarding the follow-up data, especially the radiological one, the entity of spinal cord damage and therefore the reversibility of the “snake eyes” sign is very difficult to verify with a post-operative MRI study, mainly for the presence of implanted materials, that could distort the signal and preclude the identification of such a modest lesion. Of our three cases, only one already underwent post-operative MRI (Case 3), showing the persistence of the snake-eyes myelopathy, despite clinical improvement. For the other two cases, only clinical follow-up is available so far. Most importantly, all three patients had a clinical benefit from surgery, despite the radiological evidence of SEA.

The MRI picture of “snake eyes” has always been described in relation to a clinical picture of myelopathy, but another relevant problem is related to those cases that have a “snake-eyes” MRI picture and are asymptomatic or pauci-symptomatic like Case 1. Low-intensity signal on T1WI is considered as a sign of advanced disease due to a significant neural tissue damage and correlates with poor post-operative neurological outcome [21]. Signal changes in T1WI usually appear with an increase in T2WI [22]. T2 hyperintensity in isolation cannot predict a worse post-operative outcome: the combination of both signal alteration on T1 and T2, and the presence of long segments with T2 hyperintensity are better correlated with negative neurological outcome after surgery [23].

For other myelopathies, it is clear that there may be signs of hyperintensity in MRI that are not related to a clinical myelopathy and in these cases it is not clear what needs to be done [24]. Unfortunately, given our small case series, the less severely affected pre-operative disability in respect of cases presented in literature and the fact that SEA is not a frequent entity presenting in a variety of heterogenous diseases, it was not possible to determine its independent prognostic value.

Nevertheless, it is widely accepted that the baseline neurological status is the strongest predictor of post-operative outcome.

In addition, older age is related to a worse outcome after surgery [16]. Other clinical factors such as body mass index and baseline severity score are not predictive of complications [25]. Duration of symptoms is not considered uniquely as a negative clinical factor associated with a negative outcome after surgery [16,25] We suggest that, from the pathophysiological point of view, a more severe cervical myelopathy and a longer duration of symptoms could associate with different histological damages in the spinal cord that could be reversible or not. Therefore, to achieve a good clinical outcome, it is mandatory to identify early clinical signs of cervical myelopathy [26]. In this regard, different outcome measures are reported in the literature regarding functional impairment (mJOA scale), disability (Nurick scale and Neck Disability Index), and generic short form health survey (SF-36 scale). Finally, new neurophysiological tests, like contact-heat evoked potentials, could be of help in early detection of cervical myelopathy; in fact, they seem to exhibit a superior sensitivity compared to somato-sensory evoked potentials in detecting spinal cord ischemia caused by compression of the anterior spinal artery. Concerning this, the application of classical neurophysiological techniques might be a limitation of this study [27].

## 5. Limitations of the Study

Our study suffers from several limitations. As for the analysis of the literature, few studies about DCM take into consideration surgical prognosis, and most of them have a suboptimal or no follow-up period. In addition, most studies presented an insufficient number of patients to reach the possibility of statistical inference on the general population. The variety of different pathologies that have been related to SEA myelopathy, an element that causes further difficulties in prognostic analysis, must also be considered. Furthermore, the term SEA is used unevenly in the scientific community and this may have led to loss of information. To partially compensate for this, a large number of synonyms for SEA have been used in the searching process, trying to cover the full range of terms used in the literature to describe this radiological sign. As for our case series we were unable to achieve optimal follow-up on all patients.

## 6. Conclusions

“Snake eyes” myelopathy represents a rare form of myelopathy with a prognosis that is generally defined as unfavorable. Its pathophysiology is still unclear, and its frequency might be greater than previously thought.

The literature review and personal experience of surgically treated cases shows that SEA represents a negative surgical prognosis sign in a minority (22–28%) of patients, but the baseline neurological status remains crucial to determine patients’ outcome.

## Figures and Tables

**Table 1:** Synthesis Without Meta-analysis (SWiM) in systematic reviews.

Methods	Item Description
Grouping studies for synthesis	(1a) There is a need to better understand the influence on surgical outcome of “snake-eyes” appearance (SEA) [13]. We grouped patients undergoing surgery of the cervical spine with description of magnetic resonance imaging (MRI)-evident SEA myelopathy. (1b) We extracted data about neurological outcome following surgical procedure and pathophysiological details that could aid in understanding the natural course of SEA.
Describe the standardized metric and transformation method used	Three studies reported data suitable for descriptive statistics. Only one study reported a precise *p*-value. Studies were classified based on whether they showed a reduction in the outcome measure, no effect or an increase in the outcome measure following antibiotic treatment.
Describe the synthesis method	No formal statistics were adopted by the lack of sufficient data about surgical outcome. Only descriptive statistics regarding the post-operative neurological status in SEA patients were reported.
Criteria used to prioritize results for summary and synthesis	Our inclusion criteria were: (1) studies with description of MRI-evident SEA myelopathy and surgery; (2) studies concerning a specific pathology related to SEA myelopathy, with patients undergoing surgical treatment; (3) studies with a clinical follow-up of surgically treated SEA patients.
Investigation of heterogeneity in reported effects	We explored heterogeneity visually using tables, by comparing the effect sizes of studies grouped according to potential effect modifiers. These included: baseline neurological status (i.e., Modified Japanese Orthopedic Association Score (mJOA score)); pathological condition linked to SEA (e.g., cervical spondylotic myelopathy, degenerative cervical myelopathy (DCM), etc.); study design (retrospective studies).
Certainty of evidence	Two review authors (L.Z. and G.S.) independently assessed the certainty of evidence (high, moderate, low, and very low) using the five GRADE considerations (risk of bias, consistency of effect, imprecision, indirectness, and publication bias) for each of the following outcomes to draw conclusions about certainty of the evidence. We resolved disagreements on certainty ratings by discussion and provided justification for decisions to downgrade or upgrade ratings using table footnotes.
Data presentation method	We reported the synthesis of results of the included studies classified by number of patients, type of the study, condition (DCM, cervical spondylotic myelopathy) and pre- and post-operative mJOA score evaluating interventions against ‘no-intervention’ control groups.
**Results**	
Reporting results	Regarding DCM, the “snake-eye” appearance was regarded as a negative prognostic factor in 144 cases. In particular, in Mizuno’s study, the improvement ratio determined by JOA score was 32.2% in SEA (mean post-operative mJOA score of 12.9), 47.1% in non snake-eye appearance (NSEA), and 50% (*p* < 0.01) in control cases in which high signal intensity was absent.
Limitation of the synthesis	The main limitations were the lack of randomized controlled trials (RCTs). Moreover, a thorough literature review shows an inconsistency of results about the prognostic significance of SEA in surgical and non-surgical patients and the pathogenetic mechanism is not completely understood and research is still ongoing.
